# Solubilization of *Trans*-Resveratrol in Some Mono-Solvents and Various Propylene Glycol + Water Mixtures

**DOI:** 10.3390/molecules26113091

**Published:** 2021-05-21

**Authors:** Mohammed Ghazwani, Prawez Alam, Mohammed H. Alqarni, Hasan S. Yusufoglu, Faiyaz Shakeel

**Affiliations:** 1 Department of Pharmaceutics, College of Pharmacy, King Khalid University, Abha 61441, Saudi Arabia; myghazwani@kku.edu.sa; 2 Department of Pharmacognosy, College of Pharmacy, Prince Sattam Bin Abdulaziz University, Al-Kharj 11942, Saudi Arabia; prawez_pharma@yahoo.com (P.A.); m.alqarni@psau.edu.sa (M.H.A.); h.yusufoglu@psau.edu.sa (H.S.Y.); 3 Department of Pharmaceutics, College of Pharmacy, King Saud University, Riyadh 11451, Saudi Arabia

**Keywords:** computational modeling, dissolution properties, Hansen solubility parameters, solubility, *trans*-resveratrol

## Abstract

This research deals with the determination of solubility, Hansen solubility parameters, dissolution properties, enthalpy–entropy compensation, and computational modeling of a naturally-derived bioactive compound *trans*-resveratrol (TRV) in water, methanol, ethanol, n-propanol, n-butanol, propylene glycol (PG), and various PG + water mixtures. The solubility of TRV in six different mono-solvents and various PG + water mixtures was determined at 298.2–318.2 K and 0.1 MPa. The measured experimental solubility values of TRV were regressed using six different computational/theoretical models, including van’t Hoff, Apelblat, Buchowski–Ksiazczak λh, Yalkowsly–Roseman, Jouyban–Acree, and van’t Hoff–Jouyban–Acree models, with average uncertainties of less than 3.0%. The maxima of TRV solubility in mole fraction was obtained in neat PG (2.62 × 10^−2^) at 318.2 K. However, the minima of TRV solubility in the mole fraction was recorded in neat water (3.12 × 10^−6^) at 298.2 K. Thermodynamic calculation of TRV dissolution properties suggested an endothermic and entropy-driven dissolution of TRV in all studied mono-solvents and various PG + water mixtures. Solvation behavior evaluation indicated an enthalpy-driven mechanism as the main mechanism for TRV solvation. Based on these data and observations, PG has been chosen as the best mono-solvent for TRV solubilization.

## 1. Introduction

**Figure 1:**
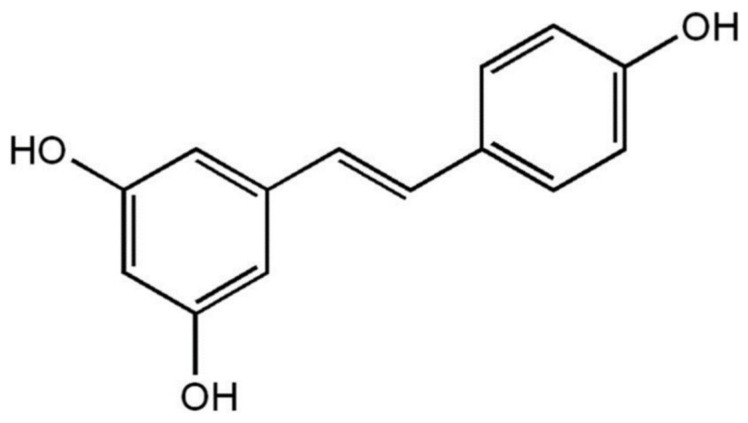
Molecular structure of *trans*-resveratrol (TRV).

*Trans*-resveratrol (TRV) {molecular structure: Figure 1; IUPAC name: 5-[(*E*)-2-(4-hydroxyphenyl) ethenyl] benzene-1,3-diol; molecular formula: C_14_H_12_O_3_; molar mass: 228.24 g mol^−1^; PubChem CID: 445154, and CAS: 501-36-0]} is a naturally-derived white crystalline powder [1,2]. It is isolated from various plant sources, such as peanuts, grapes, and berries [2,3]. TRV has shown multiple intracellular targeting properties [4]. Because of its multiple intracellular targeting properties, it has shown different therapeutic activities, such as antioxidant, cardiovascular, anti-inflammatory, neuroprotective, antidiabetic, and anticarcinogenic properties [5,6,7,8,9,10,11].

Nevertheless, its therapeutic effects are limited after oral administration because of its poor solubility in water, instability in physiological fluids, short plasma half-life, and extensive first-pass metabolism, despite its high permeability [12,13].

Various formulation strategies, such as cyclodextrin complexation [14], polymeric microparticles [15], polymeric nanoparticles [16,17], nanosponges [18], nanostructured lipid carriers [19], polymeric micelles [20], self-emulsifying drug delivery systems [21,22], self-microemulsifying drug delivery systems [23], and nanosuspensions [24] have been studied and reported to enhance solubility, dissolution, and bioavailability of TRV. The solubility values of poorly water-soluble, naturally-derived bioactive compounds in various mono-solvents and cosolvent mixtures are important for research and development with respect to their purification, crystallization, isolation/extraction from different plant sources and formulation of final drug products [1,2,25,26]. The solubility of TRV in eleven mono-solvents, such as water, methanol, ethanol, 1-propanol, 2-propanol, 1-butanol, 1-pentanol, 1-hexanol, ethyl acetate, and acetone at 278.2–318.2 K has been reported [27]. The solubility of TRV in various ethanol + water and acetone + water cosolvent mixtures at 273.2–323.2 K has also been reported [1]. The solubility of TRV in various transcutol + water cosolvent mixtures at 288.15–313.15 K has also been reported [28]. Recently, the solubility of TRV in various methanol + dichloromethane, ethanol + dichloromethane, n-propanol + dichloromethane, and n-butanol + dichloromethane cosolvent mixtures at 283.15–303.15 K has been reported [2]. The solubility of TRV in neat propylene glycol (PG) at 298.2 K and 310.2 K has also been found in the literature [22,23]. However, the solubility values, dissolution properties, and enthalpy–entropy compensation study of TRV in various PG + water mixtures have not yet been studied or reported. The vital role of PG in the solubilization of various poorly water-soluble compounds has been demonstrated well in previous studies [29,30,31].

Therefore, this research deals with the determination of solubility, Hansen solubility parameters, dissolution properties, and enthalpy–entropy compensation analysis of TRV in six mono-solvents (water, methanol, ethanol, n-propanol, n-butanol, and PG) and various PG + water mixtures at 298.2–318.2 K and laboratory atmopspheric pressure (0.1 MPa). The solubility data and other important physicochemical properties of TRV recorded in this work can be useful in the purification, crystallization, and isolation/extraction of TRV from different plant sources, as well as the formulation of final drug products.

## 2. Results and Discussion

### 2.1. Experimental Solubility of TRV and Literature Comparison

The generated experimental solubility (*x*_e_) values of TRV in water, methanol, ethanol, n-propanol, n-butanol, and PG at 298.2–318.2 K and 0.1 MPa are presented in Table 1. The generated solubility values of TRV in various PG + water mixtures at 298.2–318.2 K and 0.1 MPa are listed in Table 2.

**Figure 2:**
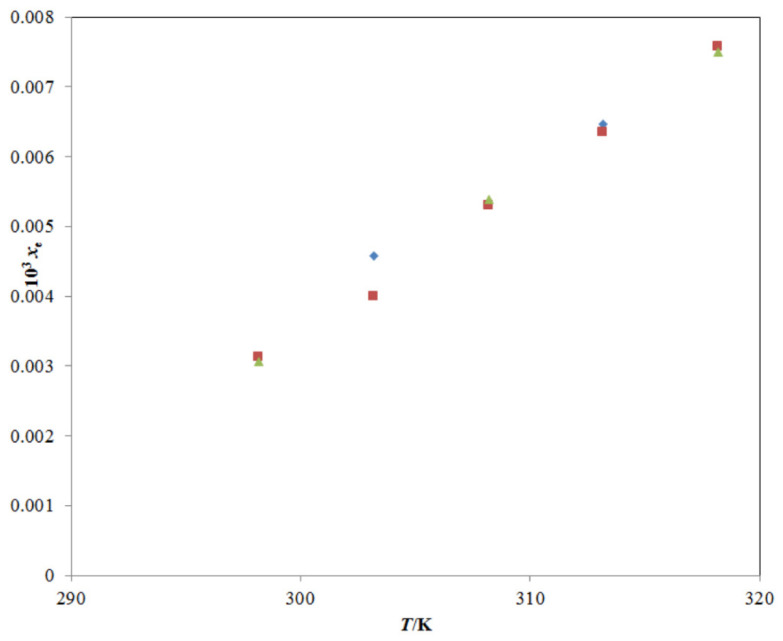
A graphical comparison of TRV solubility in neat water with reported values at different temperatures; the symbol 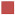 represents the experimental TRV solubility in neat water; the symbol 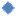 represents the reported solubility values of TRV in neat water taken from reference [27]; the symbol 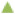 represents the reported solubility values of TRV in neat water taken from reference [1].

**Figure 3:**
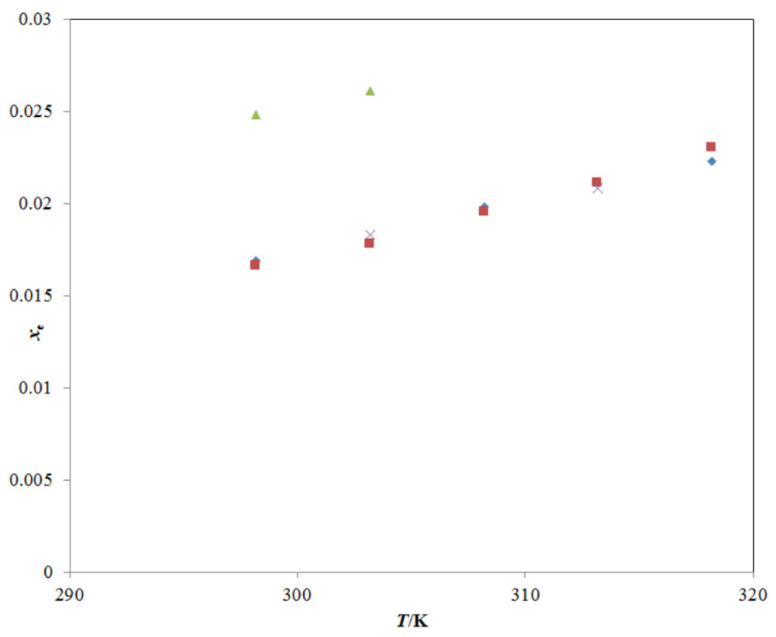
A graphical comparison of TRV solubility in neat ethanol with reported values at different temperatures; the symbol 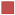 represents the experimental TRV solubility in neat ethanol; the symbol 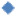 represents the reported solubility values of TRV in neat ethanol taken from reference [27]; the symbol 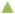 represents the reported solubility values of TRV in neat ethanol taken from reference [1]; the symbol 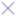 represents the reported solubility values of TRV in neat ethanol taken from reference [2].

The solubility of TRV in all studied mono-solvents at different temperatures has been reported [22,23,24]; however, the solubility and other important physicochemical parameters of TRV in various PG + water mixtures have not been reported. The mole fraction solubility of TRV in neat water at different temperatures has been reported well in the literature [1,27], and a comparison with the present experimental TRV solubility in neat water is presented in Figure 2. The experimental solubility of TRV in water was observed in good agreement with those reported in the literature [1,27]. The mole fraction solubility of TRV in neat ethanol at different temperatures has also been reported well in the literature [1,2,27], and a comparison with the present experimental TRV solubility in neat ethanol is presented in Figure 3.

**Figure 4:**
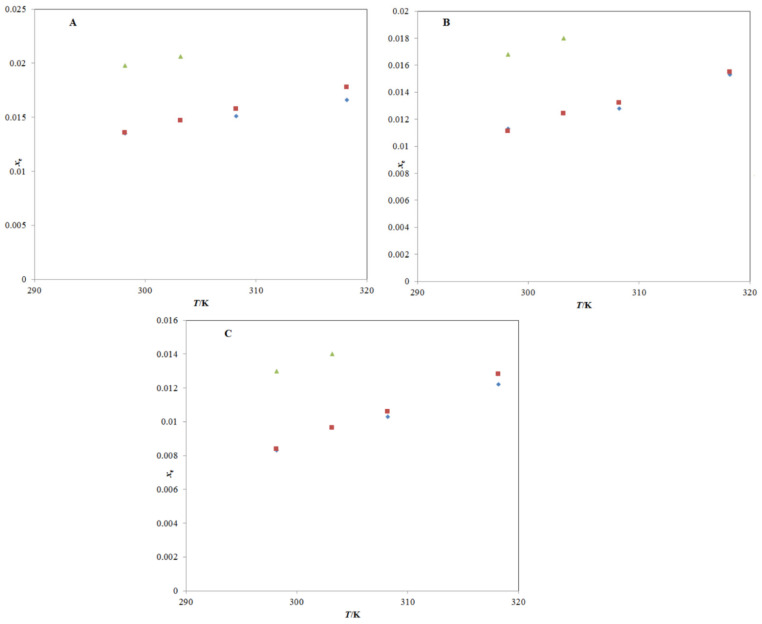
A graphical comparison of TRV solubility in (**A**) neat methanol, (**B**) neat n-propanol, and (**C**) neat n-butanol with its reported values at different temperatures; the symbol 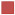 represents the experimental solubility of TRV in (**A**) neat methanol, (**B**) neat n-propanol, and (**C**) neat n-butanol; the symbol 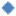 represents the reported solubility values of TRV in (**A**) neat methanol, (**B**) neat n-propanol, and (**C**) neat n-butanol taken from reference [27]; the symbol 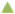 represents the reported solubility values of TRV in (**A**) neat methanol, (**B**) neat n-propanol, and (**C**) neat n-butanol taken from reference [2].

The experimental solubility of TRV in neat ethanol agreed with those reported by Sun et al. [1,27]; however, it deviated significantly from those reported by Ha et al. [2]. The mole fraction solubility of TRV in neat methanol, neat n-propanol, and neat n-butanol at different temperatures has also been reported in the literature [2,27], and a comparison with the present experimental TRV solubility in the same is presented in Figure 4A–C. The experimental solubility of TRV in neat methanol, neat n-propanol, and neat n-butanol also agreed with those reported by Sun et al. [27]; however, it deviated significantly from those reported by Ha et al. [2]. The saturated solubility of TRV in PG was reported as 63.96 mg mL^−1^ (converted to 2.16 × 10^−2^ in mole fraction) at 310.2 K by Tang et al. [23]. The saturated solubility of TRV in PG was reported as 9.22 mg mL^−1^ (converted to 3.18 × 10^−3^ in mole fraction) at 298.2 K by Balata et al. [22]. The mole fraction solubility of TRV in PG at 310.2 K was not measured in this study. The mole fraction solubility of TRV in PG at 310.2 K was obtained from the interpolation of the graph plotted between ln *x*_e_ and 1/*T* and determined as 2.13 × 10^−2^, which was much closer to those reported by Tang et al. [23]. In this work, the mole fraction solubility of TRV in PG at 298.2 K was 1.73 × 10^−2^, which deviated from those reported by Balata et al. [22]. Overall, the experimental solubilities of TRV agreed with those reported by Sun et al. and Tang et al. [1,23,27] and deviated from those reported by Ha et al. and Balata et al. [2,22]. The differences in TRV solubilities might be due to differences in analytical methods, equilibrium time, rotational speeds, and other experimental conditions.

In general, it was observed that the *x*_e_ values of TRV enhanced significantly with an increase in temperature in the mono-solvents and PG + water mixtures (*p* < 0.05) and were in accordance with previous studies [25,26,27]. Among the mono-solvents, the *x*_e_ values of TRV at 318.2 K were maximal in PG (2.62 × 10^−2^), followed by ethanol (2.30 × 10^−2^), methanol (1.77 × 10^−2^), n-propanol (1.55 × 10^−2^), n-butanol (1.25 × 10^−2^), and water (7.58 × 10^−6^). Similar data were also noted at 298.2 K, 303.2 K, 308.2 K, and 313.2 K. The solubility of TRV in all studied alcohols was of a similar magnitude at all temperatures studied (*p* > 0.05). In addition, the TRV solubility in the alcohols was significantly high compared to its solubility in water (*p* < 0.05). The solubility of the solute in different mono-solvents depends on the dynamics and polarity of the mono-solvents. The different solubility values of TRV in different mono-solvents were possible due to differences in the dynamics and polarity of the solvents [32]. The high solubility of TRV in alcohols was possible due to high intermolecular interactions between TRV and studied alcoholic mono-solvents compared to the intramolecular interactions between TRV–TRV and solvent–solvent molecules. Among different mono-solvents studied, the maximum TRV solubility was recorded in PG. Therefore, PG has been considered as the best mono-solvent for TRV solubilization. The maximum solubility of TRV in PG was possible due to higher intermolecular interactions between TRV and PG compared to those that occurred between TRV and other alcohols.

**Figure 5:**
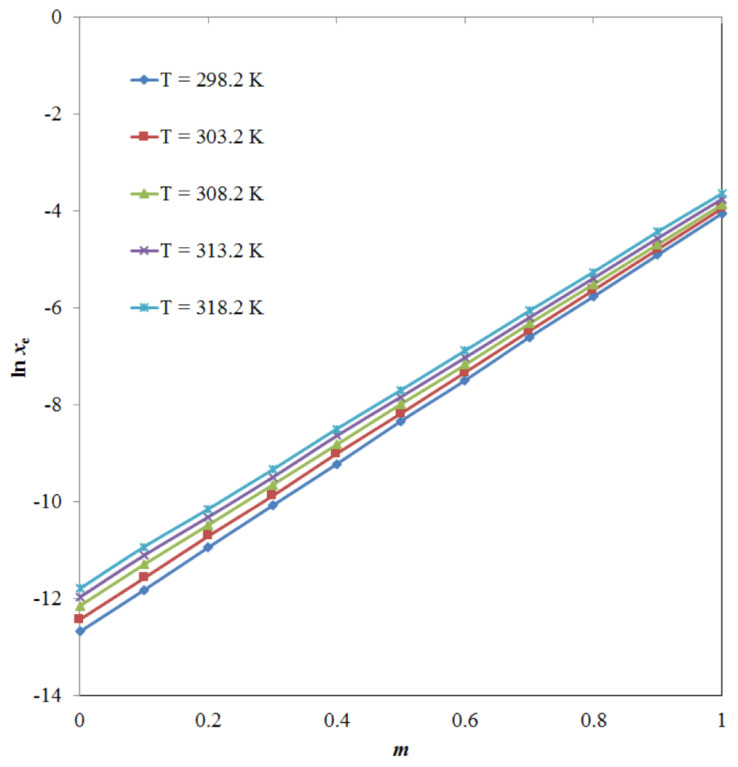
The effect of the propylene glycol (PG) mass fraction (*m*) on logarithmic solubility (ln *x*_e_) values of TRV at five different temperatures.

Among the PG + water mixtures, the *x*_e_ values of TRV were recorded at a maximum of PG mass fraction (*m*) = 0.9. Overall, TRV was soluble in all studied alcoholic mono-solvents and practically insoluble in water. The effect of the PG *m* value on the logarithmic solubility of TRV was also studied at different temperatures, and the results are shown in Figure 5. The logarithmic solubility of TRV increased significantly with an increase in the PG mass fraction (*p* < 0.05).

### 2.2. Determination of Hansen Solubility Parameters (HSPs)

The HSPs are important for the validation of experimental solubility data in order to find out the best solvent for the solubilization of materials. Therefore, different HSPs for TRV and the six mono-solvents were determined by the HSPiP software, and their predicted values are presented in Table 3. The TRV total HSP (*δ*) value was 27.10 MPa^1/2^, indicating that TRV had a medium polarity. Some of the alcoholic mono-solvents, such as methanol (*δ* = 30.30 MPa^1/2^), ethanol (*δ* = 25.40 MPa^1/2^), and PG (*δ* = 29.20 MPa^1/2^), had a *δ* value close to that of TRV and hence were suitable for TRV solubilization. The *δ* value for water was 47.80 MPa^1/2^, indicating that water is a highly polar mono-solvent and not suitable for TRV solubilization. The HSPs for various PG + water mixtures free of TRV (*δ*_mix_) are listed in Table S1. The *δ*_mix_ values for various PG + water mixtures free of TRV were 31.06–45.94 MPa^1/2^. The TRV *δ* value (27.10 MPa^1/2^) was closer to *m* = 0.9 of PG (*δ*_mix_ = 31.06 MPa^1/2^) in the PG + water mixtures, compared to other cosolvent mixtures studied. The maximum TRV experimental solubility was also obtained at *m* = 0.9 of PG in the PG + water mixtures. The calculated values of the Krevelen–Hoftyzer solubility parameter ($\Delta \bar{\delta }$), the three-dimensional solubility parameter space (*R*_a_), and Greenhalgh’s solubility parameter (∆*δ*) are also listed in Table 3. It was found that the miscibility between the solute and mono-solvent is maximal if $\Delta \bar{\delta }$ is smaller than 5.0 MPa^1/2^ [33,34]. The $\Delta \bar{\delta }$ in water ($\Delta \bar{\delta }$ = 28.26 MPa^1/2^) and methanol ($\Delta \bar{\delta }$ = 7.96 MPa^1/2^) was ≥5.0 MPa^1/2^, suggesting the immiscibility/insolubility of TRV in water and methanol, according to this concept. However, the $\Delta \bar{\delta }$ in ethanol ($\Delta \bar{\delta }$ = 4.84 MPa^1/2^), n-propanol ($\Delta \bar{\delta }$ = 4.76 MPa^1/2^), n-butanol ($\Delta \bar{\delta }$ = 4.85 MPa^1/2^), and PG ($\Delta \bar{\delta }$ = 6.86 MPa^1/2^) was much closer to <5.0 MPa^1/2^, indicating the solubility/miscibility of TRV in ethanol, n-propanol, n-butanol, and PG, according to this concept. It was also reported that the solubility between the solute and mono-solvent is maximal if *R*_a_ is smaller than 5.6 MPa^1/2^ [34,35]. The *R*_a_ was higher in all mono-solvents, suggesting the insolubility of TRV in all mono-solvents, according to this concept. The solubility between the solute and mono-solvent is maximal if ∆*δ* is smaller than 7.0 MPa^1/2^. The ∆*δ* value was maximal in water (∆*δ* = 20.70 MPa^1/2^), suggesting the insolubility of TRV in water. However, the ∆*δ* was lower in methanol (∆*δ* = 3.20 MPa^1/2^), ethanol (∆*δ* = 1.70 MPa^1/2^), n-propanol (∆*δ* = 4.20 MPa^1/2^), n-butanol (∆*δ* = 4.20 MPa^1/2^), and PG (∆*δ* = 2.10 MPa^1/2^), suggesting the miscibility of TRV in all these mono-solvents, according to this concept [36]. Overall, methanol, ethanol, and PG were suitable for TRV solubilization.

### 2.3. Computational/Theoretical Models

**Figure 6:**
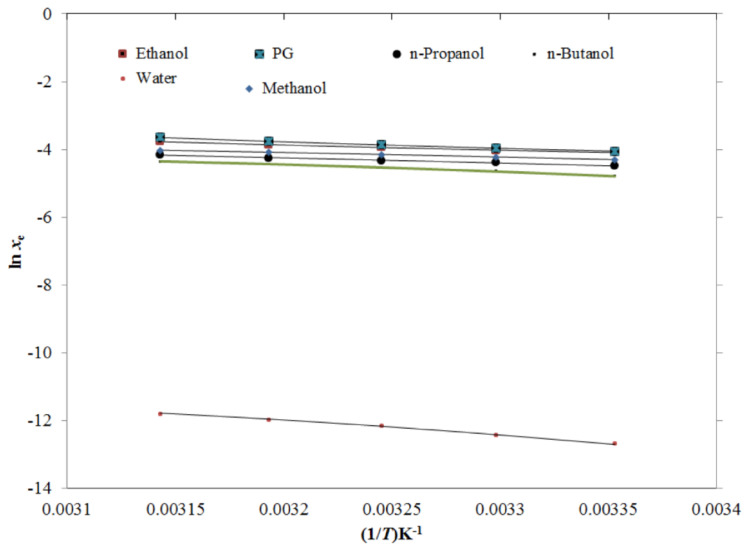
The correlation of ln *x*_e_ values of TRV with the modified Apelblat model in six different mono-solvents as a function of 1/*T*; symbols represent the experimental solubilities of TRV, and solid lines represent the solubilities of TRV calculated using the modified Apelblat model.

**Figure 7:**
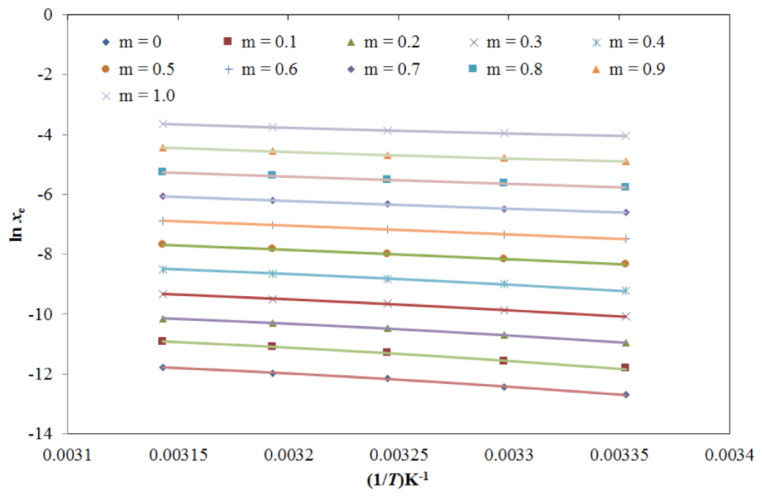
The correlation of ln *x*_e_ values of TRV with the modified Apelblat model in various PG + water compositions as a function of 1/*T*; symbols represent the experimental solubilities of TRV, and solid lines represent the solubilities of TRV calculated using the modified Apelblat model.

The generated TRV solubility data was fitted with van’t Hoff, modified Apelblat, Buchowski–Ksiazczak *λh*, Yalkowsky–Roseman, Jouyban–Acree, and Jouyban–Acree–van’t Hoff models [37,38,39,40,41,42,43,44,45]. The results of the graphical fitting between the *x*_e_ and the modified Apelblat solubility (*x*^Apl^) data of TRV in the mono-solvents as a function of 1/*T* are shown in Figure 6, which presents a good fitting between the *x*_e_ and *x*^Apl^ data of TRV in all six mono-solvents. The results for the modified Apelblat model fitting are shown in Table 4. The root mean square deviations (*RMSD*s) for TRV in the mono-solvents were estimated as 0.41%–1.71%, with an average *RMSD* of 0.89%. The TRV determination coefficient (*R*^2^) in the mono-solvents was predicted as 0.9935–0.9999. The results of the graphical fitting between the *x*_e_ and *x*^Apl^ values of TRV in the PG + water mixtures as a function of 1/*T* for the modified Apelblat model are shown in Figure 7, which also shows a good curve fitting. The results for the modified Apelblat model fitting of TRV in the PG + water mixtures are shown in Table 5. The *RMSD*s for TRV in the PG + water mixtures were estimated as 0.25%–1.87%, with an average *RMSD* of 0.93%. The TRV *R*^2^ in the PG + water mixtures was predicted as 0.9971–0.9999. These data and observations showed a good correlation of experimental solubility values of TRV in the mono-solvents and PG + water mixtures with the modified Apelblat model.

The results for the graphical fitting between the *x*_e_ and van’t Hoff model solubility (*x*^van’t^) values of TRV in the mono-solvents as a function of 1/*T* for the van’t Hoff model are shown in Figure S1, which shows a good curve fitting. The resulting data for the van’t Hoff model fitting of TRV in the mono-solvents are listed in Table 6. The *RMSD*s for TRV in the mono-solvents were 0.52%–2.67%, with an average *RMSD* of 1.35%. The TRV *R*^2^ in the mono-solvents was predicted as 0.9928–0.9980. The results for the graphical fitting between *x*_e_ and *x*^van’t^ values of TRV in the PG + water mixtures as a function of 1/*T* for the van’t Hoff model are shown in Figure S2, which also shows a good correlation.

The resulting data for the van’t Hoff model fitting of TRV in the PG + water mixtures are summarized in Table 7. The *RMSD*s for TRV in the PG + water mixtures were 0.53%–2.65%, with an average *RMSD* of 1.41%. The TRV *R*^2^ in the PG + water mixtures was predicted as 0.9935–1.0000. These data and observations again showed a good correlation of experimental solubility data of TRV in the mono-solvents and PG + water mixtures with the van’t Hoff model.

The resulting data for the Buchowski–Ksiazczak *λh* model fitting of TRV in the mono-solvents are summarized in Table 8. The *RMSD*s for TRV in the mono-solvents were estimated as 1.71%–3.14%, with an average *RMSD* of 2.37%.

The resulting data for the Buchowski–Ksiazczak *λh* model fitting of TRV in the PG + water mixtures are listed in Table 9. The *RMSD*s for TRV in the PG + water mixtures were estimated as 0.74%–3.84%, with an average *RMSD* of 2.22%. These data and observations showed a good correlation of experimental solubility data of TRV in the mono-solvents and PG + water mixtures with the Buchowski–Ksiazczak *λh* model.

The resulting data for the Yalkowsky–Roseman model fitting of TRV in the PG + water mixtures are listed in Table 10. The *RMSD*s for the Yalkowsky–Roseman model were estimated as 0.90%–3.06%, with an average *RMSD* of 1.86%.

The resulting data for the Jouyban–Acree and Jouyban–Acree–van’t Hoff model fittings of TRV in the PG + water mixtures are listed in Table 11. An overall *RMSD* for the Jouyban–Acree model was predicted as 0.82%; however, an overall *RMSD* for the Jouyban–Acree–van’t Hoff model was predicted as 0.96%. Generally, all six theoretical models performed well in the solubility correlation of TRV.

### 2.4. Dissolution Properties

Three different apparent thermodynamic quantities, including apparent standard enthalpy (Δ_sol_*H*^0^), apparent standard Gibbs energy (Δ_sol_*G*^0^), and apparent standard entropy (Δ_sol_*S*^0^), were determined to evaluate the dissolution properties of TRV. For the determination of these quantities, only the ideality of the solution was considered, and therefore, these qualities are called apparent thermodynamic quantities. The values of Δ_sol_*H*^0^ for TRV in the mono-solvents and PG + water mixtures were determined from the van’t Hoff graphs. The van’t Hoff plots for the determination of the Δ_sol_*H*^0^ values for TRV in the mono-solvents are shown in Figure S3; however, the van’t Hoff plots for the determination of the Δ_sol_*H*^0^ values for TRV in the PG + water mixtures are displayed in Figure S4. The resulting values of different thermodynamic parameters for TRV in the mono-solvents are portrayed in Table 12.

The Δ_sol_*H* values for TRV in the mono-solvents were estimated as 10.74–35.39 kJ mol^−1^. The values of Δ_sol_*G*^0^ for TRV in the mono-solvents were estimated as 9.86–31.24 kJ mol^−1^. The Δ_sol_*G*^0^ for TRV was minimal in PG and maximal in water, which may be because of the maximum and minimum solubility of TRV in PG and water, respectively. The recorded positive values of Δ_sol_*H*^0^ for TRV indicated an endothermic dissolution of TRV in all six mono-solvents [29,37]. The values of Δ_sol_*S*^0^ for TRV in the mono-solvents were also determined as positive values, in the range of 0.32–20.49 J mol^−1^ K^−1^, indicating an entropy-driven dissolution of TRV in all six mono-solvents [29]. The resulting data for the dissolution properties of TRV in the PG + water mixtures are portrayed in Table 13.

The Δ_sol_*H* values for TRV in the PG + water mixtures were estimated as 18.75–35.68 kJ mol^−1^. The Δ_sol_*G*^0^ values for TRV in the PG + water mixtures were estimated as 11.97–29.04 kJ mol^−1^. The recorded positive values of Δ_sol_*H*^0^ for TRV also suggested an endothermic dissolution of TRV in the PG + water mixtures [25,26]. The Δ_sol_*S*^0^ values for TRV in the PG + water mixtures were estimated as 14.29–22.02 J mol^−1^ K^−1^, indicating an entropy-driven dissolution of TRV in PG + water mixtures [25]. Based on all these observations and results, the dissolution of TRV was observed as endothermic and entropy-driven in the mono-solvents and PG + water mixtures [25,26,29,37].

### 2.5. Enthalpy–Entropy Compensation Evaluation

**Figure 8:**
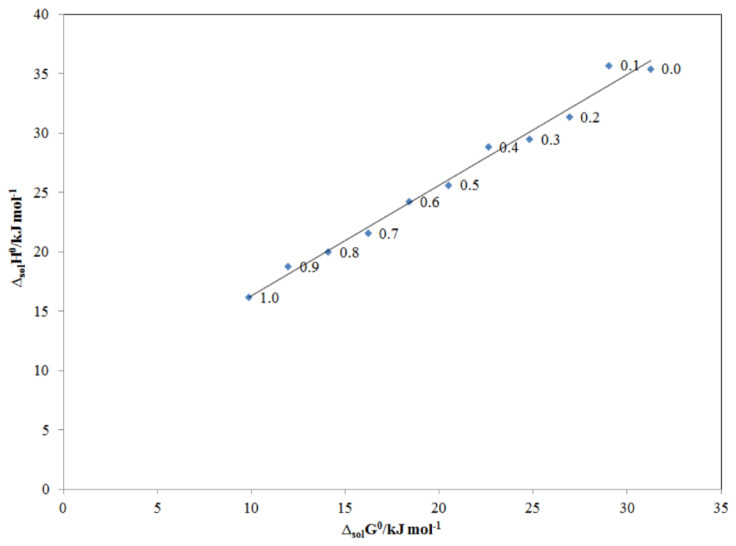
Δ_sol_*H*^0^ vs. Δ_sol_*G*^0^ enthalpy–entropy compensation plot for the solubility of TRV in various PG + water compositions at *T*_hm_ = 308 K.

The enthalpy–entropy compensation analysis is a much-debated phenomenon, which is being applied in the evaluation of thermodynamic analysis of drugs/pharmaceuticals, proteins, ligands, and nucleic acids [46,47]. It has been thoroughly evaluated using both experimental and theoretical approaches in order to understand the molecular recognition and drug design [47,48]. Therefore, enthalpy–entropy compensation analysis was applied in this work to evaluate the solvation behavior/cosolvent mechanism for TRV in the PG + water mixtures. The results of the enthalpy–entropy compensation analysis are displayed in Figure 8. TRV in all PG + water mixtures and mono-solvents (PG and water) presented a linear Δ_sol_*H*° vs. Δ_sol_*G*° graph with a positive slope value greater than unity and an *R*^2^ value greater than 0.99. Based on these observations, the driving mechanism for TRV solvation is proposed as enthalpy-driven in all PG + water mixtures, including mono-solvents (PG and water). The enthalpy-driven mechanism for TRV was possible because of the higher solvation of TRV in PG molecules compared to its solvation in water molecules [49,50]. It is well-known that water helps in determining the stability, structure, dynamics, and functions of hydrophilic as well as hydrophobic molecules [51]. In this work, TRV/water solvation was much weaker than TRV/PG solvation. This observation was due to the fact that the intermolecular interactions between TRV–PG molecules were higher compared to the intramolecular interactions between TRV–TRV and water–water molecules. The recorded solvation behavior of TRV in the PG + water mixtures was similar to those proposed for the solvation behavior of vanillin and sulfacetamide in various PG + water mixtures [29,31].

## 3. Materials and Methods

### 3.1. Materials

TRV (mass fraction purity = 0.993) was obtained from Sigma-Aldrich (St. Louis, MO, USA). The mono-solvents of analytical grades, such as methanol, ethanol, n-propanol, and n-butanol, were obtained from Alfa Aesar (Ward Hill, MA, USA). PG of analytical grade was obtained from E-Merck (Darmstadt, Germany). The chromatography grade/deionized water (specific conductivity <1.0 µS cm^−1^ and pH 6.7) was obtained from an ELGA water purifier (ELGA, Wycombe, UK). Further purification was not performed because all materials were of high purity. The details of the TRV and mono-solvents, such as source, molecular formula, molar mass, purification method, method of analysis, and the CAS number, are summarized in Supplementary Table S2.

### 3.2. Preparation of PG + Water Solvent Mixtures

All PG + water solvent mixtures were obtained on a mass basis, using a Digital Analytical Balance (Mettler Toledo, Greifensee, Switzerland) with a sensitivity of ± 0.10 mg. The mass fraction of PG for different PG + water solvent mixtures was obtained, varied by 0.10, from 0.10–0.90. Each PG + water solvent mixture was obtained in triplicate.

### 3.3. Measurement of TRV Solubility

The TRV solubility in water, methanol, ethanol, n-propanol, n-butanol, PG, and binary PG + water mixtures was measured using a shake flask technique at 298.2–318.2 K and 0.1 MPa [52]. The same experimental conditions were applied as those explained in our previous studies [26,29]. The amount of TRV in equilibrium samples of six different mono-solvents and various PG + water mixtures was determined by a reported high-performance liquid chromatography (HPLC) method at 306 nm [53]. The ternary mixture of methanol: 10 mM potassium dihydrogen phosphate buffer (pH 6.8): acetonitrile (63:30:7, *v*/*v*/*v*) was utilized as the mobile phase for HPLC determination of TRV. The amount of TRV (μg g^−1^) was obtained using a TRV calibration curve. The *x*_e_ value of TRV in the mono-solvents and PG + water mixtures were calculated using their standard equations, reported previously [26,29].

### 3.4. Determination of HSPs

The HSPs are generally employed to estimate the miscibility of solute in a mono-solvent or cosolvent mixtures. Hansen had separated total HSP (*δ*) into three contributions, including hydrogen bonding (*δ*_h_), dipole interactions (*δ*_p_), and non-polar interactions (*δ*_d_). It has been generally assumed that if the HSP of solute and mono-solvent/ solvent mixtures are similar, it will give the maximum solubility [54,55]. Therefore, various HSPs for TRV, water, methanol, ethanol, n-propanol, n-butanol, PG, and various PG + water mixtures free of TRV were calculated in this work, using Equation (1) [36,37,38]:

(1) $$\delta^{2}=\delta_{d}^{2}+\delta_{p}^{2}+\delta_{h}^{2}$$

The values of *δ*, *δ*_d_, *δ*_p_, and *δ*_h_ for TRV and various mono-solvents were obtained using HSPiP software (version 4.1.07, Louisville, KY, USA) by putting the simplified molecular-input line-entry system (SMILES) of TRV and six different mono-solvents. The SMILES for each component were taken directly from the PubChem database.

The HSPs for different PG + water mixtures (*δ*_mix_) free of TRV were determined using Equation (2) [54]:

(2) $$\delta_{\mathrm{mix}}=\propto \delta_{1}+\left(1-\propto \right)\delta_{2}$$

where *α* is the PG volume fraction in PG + water mixtures; *δ*_1_ is the PG HSP; *δ*_2_ is the water HSP.

The Van Krevelen–Hoftyzer method confirmed the miscibility of solute and solvent using the $\Delta \bar{\delta }$. The $\Delta \bar{\delta }$ between TRV and a specific mono-solvent was determined using Equation (3) [33,55]:

(3) $$\Delta \bar{\delta }=\left(\delta_{d}{}_{2}^{2}-\delta_{d}{}_{1}^{2}\right)+\left(\delta_{p}{}_{2}^{2}-\delta_{p}{}_{1}^{2}\right)+{\left(\delta_{h}{}_{2}^{2}-\delta_{h}{}_{1}^{2}\right)}^{1/2}$$

where subscripts 1 and 2 refer to the specific mono-solvent and TRV, respectively. It was evidenced that the maximum solubility/miscibility between the solute and mono-solvent is attained if $\Delta \bar{\delta }$ is smaller than 5.0 MPa^1/2^ [37,55]. The *R*_a_ between TRV and the mono-solvent was determined using Equation (4) [34,56]:

(4) $$Ra^{2}=4{\left(\delta_{d2}-\delta_{d1}\right)}^{2}+{\left(\delta_{p2}-\delta_{p1}\right)}^{2}+{\left(\delta_{h2}-\delta_{h1}\right)}^{2}$$

Based on *R*_a_ data, the maximum solubility between the solute and mono-solvent can be attained if *R*_a_ is smaller than 5.6 MPa^1/2^ [34,37]. However, higher *R*_a_ values correspond to poor solubility between the solute and mono-solvent.

The ∆*δ* between TRV and the mono-solvent was obtained using Equation (5) [36]:

(5) $$\Delta \delta =\delta_{2}-\delta_{1}$$

where subscripts 1 and 2 refer to the specific mono-solvent and TRV, respectively. According to this theory, the solute and mono-solvent are miscible if ∆*δ* is smaller than 7.0 MPa^1/2^, whereas the combination of the solute and mono-solvent is immiscible if ∆*δ* is greater than 10.0 MPa^1/2^ [36].

### 3.5. Computational/Theoretical Models

The generated TRV solubility data in six mono-solvents and various PG + water mixtures were fitted using six theoretical models. The TRV solubility in the six mono-solvents was fitted using van’t Hoff, modified Apelblat, and Buchowski–Ksiazczak *λh* models [37,38,39,40,41], while the TRV solubility in various PG + water mixtures were fitted with van’t Hoff, modified Apelblat, Buchowski-Ksiazazak *λh*, Yalkowsky–Roseman, Jouyban–Acree, and Jouyban–Acree–van’t Hoff models [37,38,39,40,41,42,43,44,45]. The *x*^Apl^ of TRV in the mono-solvents and mixtures was determined using Equation (6) [38,39]:

(6) $$\ln\;x^{\mathrm{Apl}}=A+\frac{B}{T}+C\ln\left(T\right)$$

where, *A*, *B*, and *C* are the coefficients of Equation (6), which were predicted using nonlinear multivariate regression analysis of the TRV experimental solubilities included in Table 1 for the mono-solvents and Table 2 for the PG + water mixtures [37]. The fitting between *x*_e_ and *x*^Apl^ of TRV was carried out in terms of *RMSD* and *R*^2^. The *RMSD* was obtained using its standard formula reported in the literature [26,29].

The *x*^van’t^ of TRV in the mono-solvents and PG + water mixtures was calculated using Equation (7) [37]:

(7) $$\ln\;x^{\mathrm{van}’t}=a+\frac{b}{T}$$

where, *a* and *b* are the parameters of the Equation (7). These parameters were obtained by the method of least squares [25].

The Buchowski–Ksiazczak *λh* model solubility (*x*) of TRV was calculated using Equation (8) [40,41]:

(8) $$\ln\;[1+\frac{\lambda \left(1-x\right)}{x}]=\lambda h\;[\frac{1}{T}-\frac{1}{T_{\mathrm{fus}}}]\;$$

where *λ* and *h* are the parameters of Equation (8).

The logarithmic solubility of the Yalkowsky–Roseman model (log *x*^Yal^) for TRV in various PG + water mixtures was calculated using Equation (9) [42]:

(9) $$\mathrm{Log}x^{\mathrm{Yal}}=m_{1}\log x_{1}+m_{2}\log x_{2}$$

where *x*_1_ is the solubility of TRV in PG; *x*_2_ is the solubility of TRV in water; *m*_1_ is the PG mass fraction; *m*_2_ is the water mass fraction.

The Jouyban–Acree model solubility (*x*_m,T_) for TRV in various PG + water mixtures was calculated by Equation (10) [43,44,45]:

(10) $$\ln\;x_{m}{,}_{T}=m_{1}\ln x_{1}+m_{2}\;\ln\;x_{2}+[\frac{m_{1\;}m_{2}}{T}\sum_{i=0}^{2}\;J_{i}(m_{1}-m_{2})]$$

where *J*_i_ is the model parameter of Equation (10), which was obtained by applying no-intercept regression analysis [43,57].

The Jouyban–Acree–van’t Hoff model solubility (*x*_m,T_) for TRV in various PG + water mixtures was calculated by Equation (11) [58,59]:

(11) $$\ln\;x_{m}{,}_{T}=m_{1}\left(A_{1}+\frac{B_{1}}{T}\right)+m_{2}\;\left(A_{2}+\frac{B_{2}}{T}\right)+\left[\frac{m_{1}m_{2}}{T}\;\sum_{i=0}^{2}J_{i}\left(m_{1}-m_{2}\right)\right]$$

where *A*_1_, *B*_1_, *A*_2_, *B*_2_, and *J_i_* are the parameters of Equation (11).

### 3.6. Dissolution Properties

For the evaluation of dissolution properties of TRV in the mono-solvents and PG + water mixtures, apparent thermodynamic analysis was conducted. Three thermodynamic parameters for TRV dissolution properties—Δ_sol_*H*^0^, Δ_sol_*G*^0^, and Δ_sol_*S*^0^—were determined [60,61,62]. The well-known van’t Hoff equation was employed to determine the Δ_sol_*H*^0^ values for TRV in the mono-solvents and PG + water mixtures using Equation (12) [60,61]:

(12) ∂ln xe∂1T−1ThmP=−ΔsolH0R

where *T*_hm_ is the mean harmonic temperature and was determined as 308 K. By plotting ln *x*_e_ values of TRV vs. 1T−1Thm, the *Δ*_sol_*H*^0^, and *Δ*_sol_*G*^0^ values for TRV were obtained from the slope and intercept, respectively, using Equations (13) and (14), respectively [60,61]:

(13) ΔsolH0=−R∂ln xe∂1T−1ThmP

(14) $$\Delta_{\mathrm{sol}}G^{0}=-RT_{\mathrm{hm}}\times \mathrm{intercept}\;$$

The *Δ*_sol_*S*^0^ values for TRV in the mono-solvents and PG + water mixtures were calculated using Equation (15) [60,61,62]:

(15) $$\Delta_{\mathrm{sol}}S^{0}=\frac{\Delta_{\mathrm{sol}}H^{0}-\Delta_{\mathrm{sol}}G^{0}}{T_{\mathrm{hm}}}\;$$

### 3.7. Enthalpy–Entropy Compensation Evaluation

The solvation behavior of TRV in the PG + water mixtures was evaluated using an enthalpy–entropy compensation analysis, detailed in previous studies [49,50]. This analysis was performed by plotting the weighted graphs of Δ_sol_*H*° and Δ_sol_*G*° at *T*_hm_ = 308 K [49].

### 3.8. Statistical Evaluation

Statistical evaluation was conducted by adopting the Kruskal–Wallis analysis followed by Denn’s test, using GraphpadInstat software (Version 9.1.1, San Diego, CA, USA). A *p*-value of less than 0.05 was taken as significant.

## 4. Conclusions

This study deals with the evaluation of solubility, HSPs, dissolution properties, enthalpy–entropy compensation, and computational modeling of a naturally-derived bioactive compound TRV in water, methanol, ethanol, n-propanol, n-butanol, PG, and various PG + water mixtures. Various HSPs were estimated to select the best mono-solvent for TRV solubility. TRV experimental solubility values were correlated well with van’t Hoff, modified Apelblat, Buchowski–Ksiazczak *λh*, Yalkowsky–Roseman, Jouyban–Acree, and Jouyban–Acree–van’t Hoff models. The TRV solubility was enhanced significantly with increased temperature (*p* < 0.05) in water, methanol, ethanol, n-propanol, n-butanol, PG, and various PG + water mixtures. The TRV solubility was maximal in PG, followed by ethanol, methanol, n-propanol, n-butanol, and water, at each temperature studied. Dissolution studies portrayed an endothermic and entropy-driven dissolution of TRV in water, methanol, ethanol, n-propanol, n-butanol, PG, and various PG + water mixtures. The enthalpy–entropy compensation evaluation suggested an enthalpy-driven mechanism as the main mechanism for TRV solvation. Based on all these data and observations, PG is the best mono-solvent for the solubilization of TRV.

## Figures and Tables

**Table 1:** Experimental solubilities (*x*_e_) of *trans*-resveratrol (TRV) in the mole fraction in six different mono-solvents at 298.2–318.2 K and 0.1 MPa ^a^.

Components	*x*_e_				
	*T* = 298.2 K	*T* = 303.2 K	*T* = 308.2 K	*T* = 313.2 K	*T* = 318.2 K
Water	3.12 × 10^−6^	4.00 × 10^−6^	5.30 × 10^−6^	6.35 × 10^−6^	7.58 × 10^−6^
Methanol	1.35 × 10^−2^	1.46 × 10^−2^	1.57 × 10^−2^	1.68 × 10^−2^	1.77 × 10^−2^
Ethanol	1.66 × 10^−2^	1.78 × 10^−2^	1.95 × 10^−2^	2.11 × 10^−2^	2.30 × 10^−2^
n-Propanol	1.11 × 10^−2^	1.24 × 10^−2^	1.32 × 10^−2^	1.42 × 10^−2^	1.55 × 10^−2^
n-Butanol	8.37 × 10^−3^	9.64 × 10^−3^	1.06 × 10^−2^	1.18 × 10^−2^	1.28 × 10^−2^
PG	1.73 × 10^−2^	1.92 × 10^−2^	2.08 × 10^−2^	2.34 × 10^−2^	2.62 × 10^−2^

^a^ The relative uncertainties *u*_r_ are *u*_r_(*T*) = 0.010, *u*(*p*) = 0.003, and *u*_r_(*x*_e_) = 0.017.

**Table 2:** The *x*_e_ values of TRV against mass fraction of propylene glycol (PG; *m*) in binary PG + water compositions at 298.2–318.2 K and 0.1 MPa ^b^.

*m*	*x*_e_				
	*T* = 298.2 K	*T* = 303.2 K	*T* = 308.2 K	*T* = 313.2 K	*T* = 318.2 K
0.1	7.35 × 10^−6^	9.40 × 10^−6^	1.25 × 10^−5^	1.50 × 10^−5^	1.79 × 10^−5^
0.2	1.77 × 10^−5^	2.24 × 10^−5^	2.81 × 10^−5^	3.32 × 10^−5^	3.91 × 10^−5^
0.3	4.20 × 10^−5^	5.12 × 10^−5^	6.43 × 10^−5^	7.50 × 10^−5^	8.80 × 10^−5^
0.4	9.88 × 10^−5^	1.23 × 10^−4^	1.48 × 10^−4^	1.77 × 10^−4^	2.04 × 10^−4^
0.5	2.40 × 10^−4^	2.81 × 10^−4^	3.410 × 10^−4^	3.96 × 10^−4^	4.54 × 10^−4^
0.6	5.56 × 10^−4^	6.55 × 10^−4^	7.65 × 10^−4^	8.87 × 10^−4^	1.03 × 10^−3^
0.7	1.35 × 10^−3^	1.54 × 10^−3^	1.79 × 10^−3^	2.03 × 10^−3^	2.33 × 10^−3^
0.8	3.13 × 10^−3^	3.57 × 10^−3^	4.04 × 10^−3^	4.58 × 10^−3^	5.20 × 10^−3^
0.9	7.41 × 10^−3^	8.30 × 10^−3^	9.19 × 10^−3^	1.05 × 10^−2^	1.20 × 10^−2^

^b^ The relative uncertainties *u*_r_ are *u*_r_(*T*) = 0.013, *u*_r_(*m*) = 0.010, *u*_r_(*p*) = 0.003 and *u*_r_(*x*_e_) = 0.020.

**Table 3:** Various solubility parameters of TRV and six different mono-solvents at 298.2 K.

Components	Hansen Solubility Parameters				*R*_a_*/MPa^1/2^	$\Delta \bar{\delta }$/MPa^1/2^	∆*δ* */MPa^1/2^
	*δ*_d_/MPa^1/2^	*δ*_p_/MPa^1/2^	*δ*_h_/MPa^1/2^	*δ*/MPa^1/2^			
TRV	20.60	7.30	15.90	27.10	-	-	-
Water	15.50	16.00	42.30	47.80	29.60	28.26	20.70
Methanol	17.40	10.60	22.40	30.30	9.70	7.96	3.20
Ethanol	16.20	8.40	17.60	25.40	9.02	4.84	1.70
n-Propanol	16.00	7.00	14.70	22.90	9.28	4.76	4.20
n-Butanol	15.90	6.30	15.20	22.90	9.47	4.85	4.20
PG	17.40	9.10	21.70	29.20	8.82	6.86	2.10

* These values were calculated between TRV and respective mono-solvents.

**Table 4:** The results of the Apelblat model for TRV in six different mono-solvents.

Components	*A*	*B*	*C*	*R* ^2^	*RMSD* (%)	Overall *RMSD* (%)
Water	762.67	−39195	−113.01	0.9969	1.71	
Methanol	152.42	−8288.9	−22.620	0.9999	0.41	
Ethanol	−186.16	7036.6	27.810	0.9989	0.51	0.89
n-Propanol	79.710	−5141.1	−11.750	0.9935	0.93	
n-Butanol	412.24	−20850	−60.920	0.9990	0.91	
PG	−287.67	11366	43.080	0.9974	0.87	

**Table 5:** The results of the Apelblat model for TRV in various PG + water compositions.

*m*	*A*	*B*	*C*	*R* ^2^	*RMSD* (%)	Overall *RMSD* (%)
0.1	718.95	−37178	−106.37	0.9971	1.87	
0.2	629.72	−32599	−93.250	0.9986	1.11	
0.3	409.21	−22250.4	−60.490	0.9977	1.36	
0.4	488.56	−25784	−72.190	0.9999	0.87	
0.5	166.97	−10657	−24.490	0.9981	1.02	0.93
0.6	45.190	−4886.7	−6.3700	0.9999	0.25	
0.7	−122.25	3109.0	18.460	0.9993	0.61	
0.8	−82.530	1488.6	12.590	0.9998	0.47	
0.9	−368.28	14764	55.080	0.9989	0.87	

**Table 6:** The results of the van’t Hoff model for TRV in six different mono-solvents.

Components	*a*	*b*	*R* ^2^	*RMSD* (%)	Overall *RMSD* (%)
Water	1.6000	−4252.1	0.9928	2.67	
Methanol	0.03000	−1290.8	0.9980	0.52	
Ethanol	1.1000	−1553.8	0.9969	1.04	1.35
n-Propanol	0.55000	−1503.3	0.9931	1.20	
n-Butanol	1.9800	−2014.7	0.9933	1.47	
PG	2.4500	−1943.0	0.9940	1.23	

**Table 7:** The results of the van’t Hoff model for TRV in various PG + water compositions.

*m*	*a*	*b*	*R* ^2^	*RMSD* (%)	Overall *RMSD* (%)
0.1	2.5700	−4286.5	0.9935	2.65	
0.2	1.7000	−3764.3	0.9950	1.98	
0.3	1.8000	−3541.2	0.9961	1.76	
0.4	2.3900	−3460.7	0.9973	1.67	
0.5	1.9700	−3075.0	0.9979	1.28	1.41
0.6	2.2500	−2907.0	1.0000	0.59	
0.7	2.0700	−2589.4	0.9990	0.53	
0.8	2.2600	−2396.0	0.9996	0.71	
0.9	2.6300	−2252.4	0.9949	1.54	

**Table 8:** The results of the Buchowski–Ksiazczak *λh* model for TRV in six different mono-solvents.

*m*	*λ*	*h*	*RMSD* (%)	Overall *RMSD* (%)
Water	5.2900	802.97	3.14	
Methanol	1.3600	946.05	1.71	
Ethanol	0.77000	1979.1	2.12	2.37
n-Propanol	1.2300	1217.9	2.24	
n-Butanol	0.75000	2673.7	2.58	
PG	0.15000	12666	2.47	

**Table 9:** The results of the Buchowski–Ksiazczak *λh* model for TRV in various PG + water compositions.

Samples	*λ*	*h*	*RMSD* (%)	Overall *RMSD* (%)
0.1	4.3800	976.58	3.84	
0.2	4.2800	877.56	3.12	
0.3	3.7700	939.26	2.82	
0.4	3.0200	1142.4	2.74	
0.5	2.7300	1124.4	2.42	2.22
0.6	2.1400	1356.0	1.04	
0.7	1.7300	1491.5	0.74	
0.8	1.1800	2025.0	0.95	
0.9	0.54000	4126.0	2.34	

**Table 10:** Log *x*^Yal^ values of TRV calculated using the Yalkowsky–Roseman model in various PG + water compositions at 298.2–318.2 K.

*m*	Log *x*^Yal^					*RMSD* (%)	Overall *RMSD* (%)
	298.15	303.15	308.15	313.15	318.15		
0.1	−5.13	−5.02	−4.91	−4.84	−4.76	3.06	
0.2	−4.75	−4.66	−4.55	−4.48	−4.41	1.55	
0.3	−4.38	−4.29	−4.19	−4.12	−4.05	0.90	
0.4	−4.00	−3.92	−3.83	−3.77	−3.70	2.80	
0.5	−3.63	−3.55	−3.47	−3.41	−3.35	2.21	1.86
0.6	−3.25	−3.18	−3.11	−3.05	−2.99	1.20	
0.7	−2.88	−2.81	−2.75	−2.70	−2.64	2.29	
0.8	−2.50	−2.45	−2.39	−2.34	−2.28	1.10	
0.9	−2.13	−2.08	−2.03	−1.98	−1.93	1.69	

**Table 11:** The results of the Jouyban–Acree and Jouyban–Acree–van’t Hoff models for TRV in PG + water mixtures.

System	Jouyban–Acree	Jouyban–Acree-Van’t Hoff
		*A*_1_ 2.45
PG + water	*J*_i_ 83.20	*B*_1_ −1943.00
		*A*_2_ 1.60
		*B*_2_ −4252.10
*RMSD* (%)		*J*_i_ 78.65
	0.82	0.96

**Table 12:** Apparent thermodynamic parameters (Δ_sol_*H*^0^, Δ_sol_*G*^0^, and Δ_sol_*S*^0^) and *R*^2^ values for TRV in six different mono-solvents ^c^.

Components	Δ_sol_*H*^0^/kJ mol^−1^	Δ_sol_*G*^0^/kJ mol^−1^	Δ_sol_*S*^0^/J mol^−1^ K^−1^	*R* ^2^
Water	35.39	31.24	13.47	0.9927
Methanol	10.74	10.64	0.32	0.9939
Ethanol	12.93	10.07	9.27	0.9970
n-Propanol	12.51	11.06	4.69	0.9930
n-Butanol	16.77	11.65	16.60	0.9931
PG	16.17	9.86	20.49	0.9941

^c^ The average relative uncertainties are *u*_r_(Δ_sol_*H*^0^) = 0.52, *u*_r_(Δ_sol_*G*^0^) = 0.59, and *u*_r_(Δ_sol_*S*^0^) = 0.69; these uncertainties are relative standard deviations of all values of each thermodynamic quantity.

**Table 13:** Apparent thermodynamic quantities (Δ_sol_*H*^0^, Δ_sol_*G*^0^, and Δ_sol_*S*^0^) and *R*^2^ values for TRV in various PG + water compositions ^d^.

Parameters	*m* = 0.1	*m* = 0.2	*m* = 0.3	*m* = 0.4	*m* = 0.5	*m* = 0.6	*m* = 0.7	*m* = 0.8	*m* = 0.9
Δ_sol_*H*^0^/kJ mol^−1^	35.68	31.33	29.48	28.81	25.60	24.20	21.55	19.94	18.75
Δ_sol_*G*^0^/kJ mol^−1^	29.04	26.93	24.81	22.62	20.50	18.39	16.21	14.11	11.97
Δ_sol_*S*^0^/J mol^−1^ K^−1^	21.53	14.29	15.15	20.07	16.55	18.86	17.33	18.94	22.02
*R* ^2^	0.9934	0.9948	0.9960	0.9972	0.9979	0.9999	0.9991	0.9997	0.9951

^d^ The average relative uncertainties are *u*_r_(Δ_sol_*H*^0^) = 0.21, *u*_r_(Δ_sol_*G*^0^) = 0.28, and *u*_r_(Δ_sol_*S*^0^) = 0.14; these uncertainties are relative standard deviations of all values of each thermodynamic quantity.

## Data Availability

All data associated with this article are included in Supplementary Materials.

## Supplementary Materials

The following are available online, Figure S1: Correlation of ln *x*_e_ values of TRV with the van’t Hoff model in six different mono-solvents as a function of 1/*T*; symbols represent the experimental solubilities of TRV, and solid lines represent the solubilities of TRV calculated using the van’t Hoff model, Figure S2: The correlation of ln *x*_e_ values of TRV with the van’t Hoff model in various PG + water compositions as a function of 1/*T*; symbols represent the experimental solubilities of TRV, and solid lines represent the solubilities of TRV calculated using the van’t Hoff model, Figure S3: van’t Hoff plots for TRV plotted between ln *x*_e_ and 1/*T*-1/*T*_hm_ for TRV in six different mono-solvents, Figure S4: van’t Hoff plots for TRV plotted between ln *x*_e_ and 1/*T*-1/*T*_hm_ for TRV in various PG + water mixtures, Table S1: Hansen solubility parameters (*δ*_mix_/MPa^1/2^) for various PG + water mixtures free of TRV at 298.2 K, Table S2: Details information about materials used.

## Sample Availability

Samples of the compounds TRV are available from the authors.
