# Supplementary material for: Solubilization of Trans-Resveratrol in Some Mono-Solvents and Various Propylene Glycol + Water Mixtures
Source: Molecules. 2021 May 21;26(11):3091. doi: 10.3390/molecules26113091 (PMC8196874; doi:10.3390/molecules26113091)
Supplement: Supplementary file 1 [file molecules-26-03091-s001.zip › molecules-1213603-supplementary.pdf]

Supplementary materials

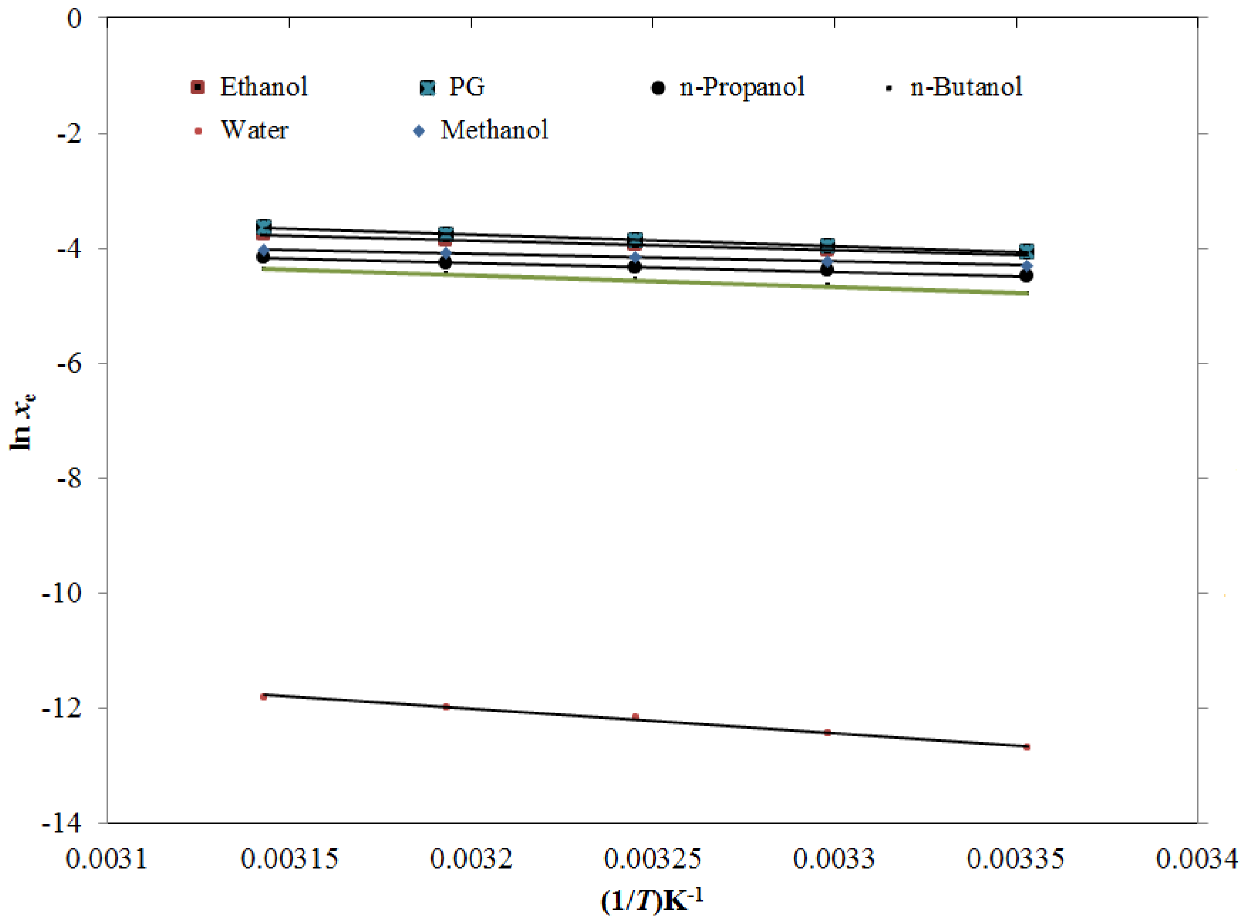

Figure S1. Correlation of ln x_e values of TRV with van't Hoff model in six different mono-solvents as a function of 1/T; symbols represent the experimental solubilities of TRV and solid lines represent the solubilities of TRV calculated using van't Hoff model.

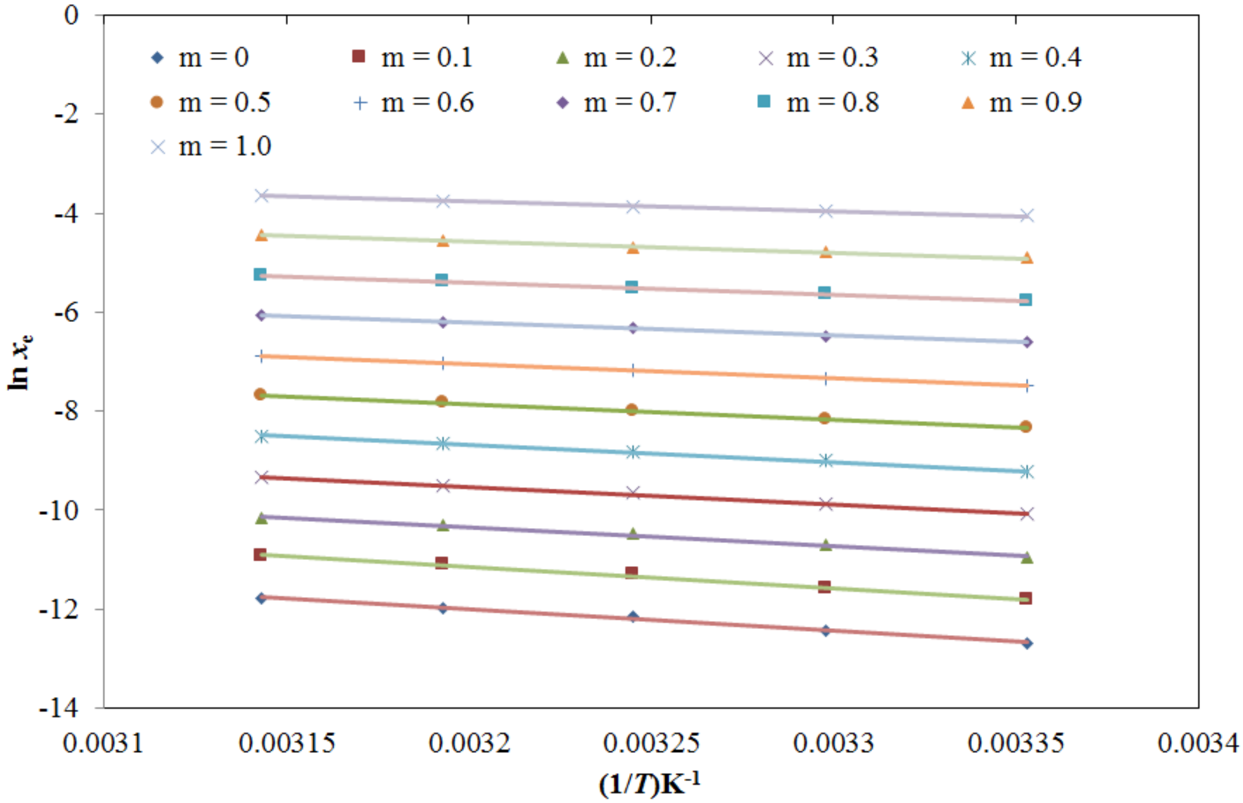

Figure S2. Correlation of ln x_e values of TRV with van't Hoff model in various PG + water compositions as a function of 1/*T*; symbols represent the experimental solubilities of TRV and solid lines represent the solubilities of TRV calculated using van't Hoff model.

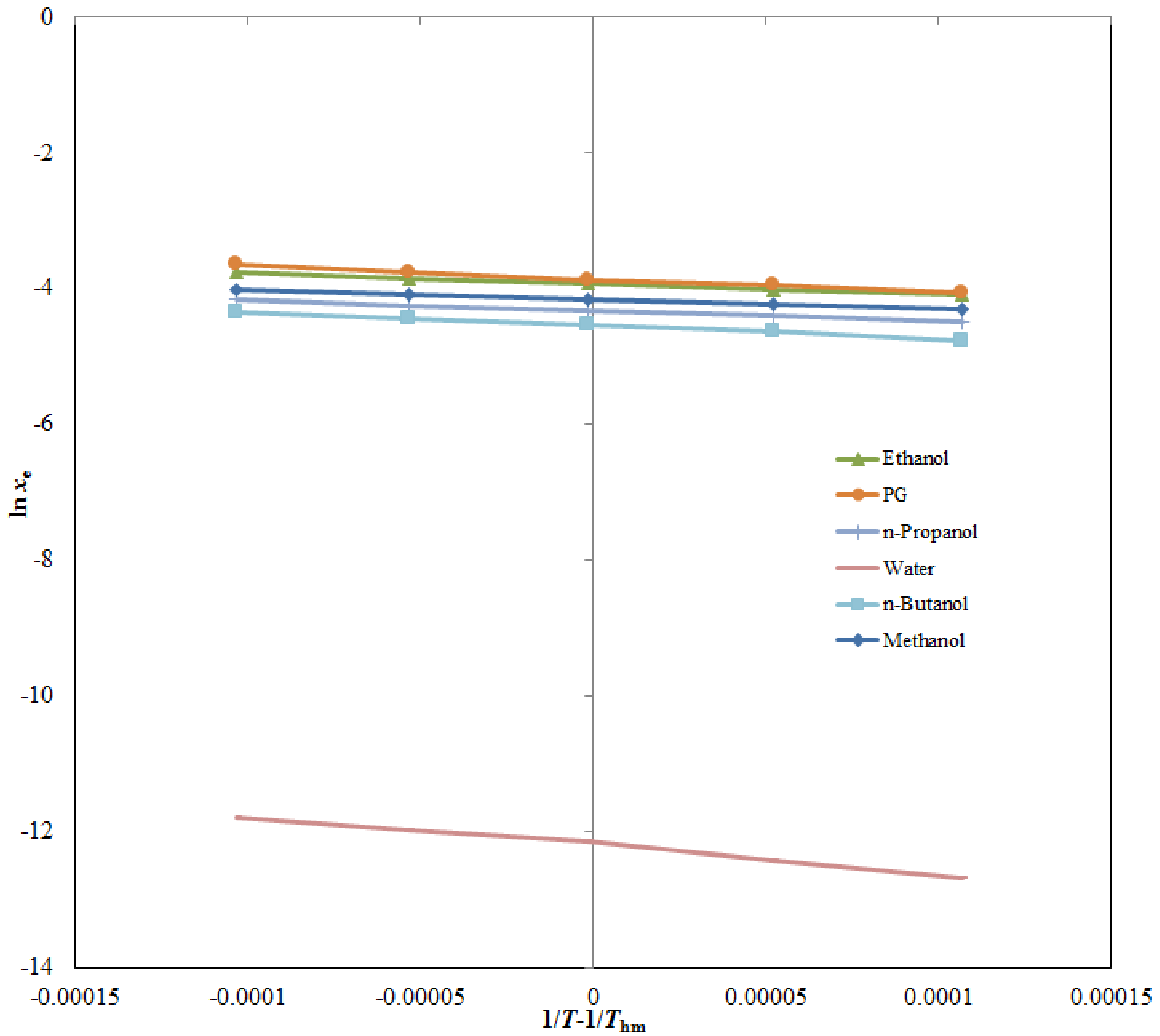

Figure S3. van't Hoff plots for TRV plotted between ln x_e and $1/T$-$1/T_{hm}$ for TRV in six different mono-solvents.

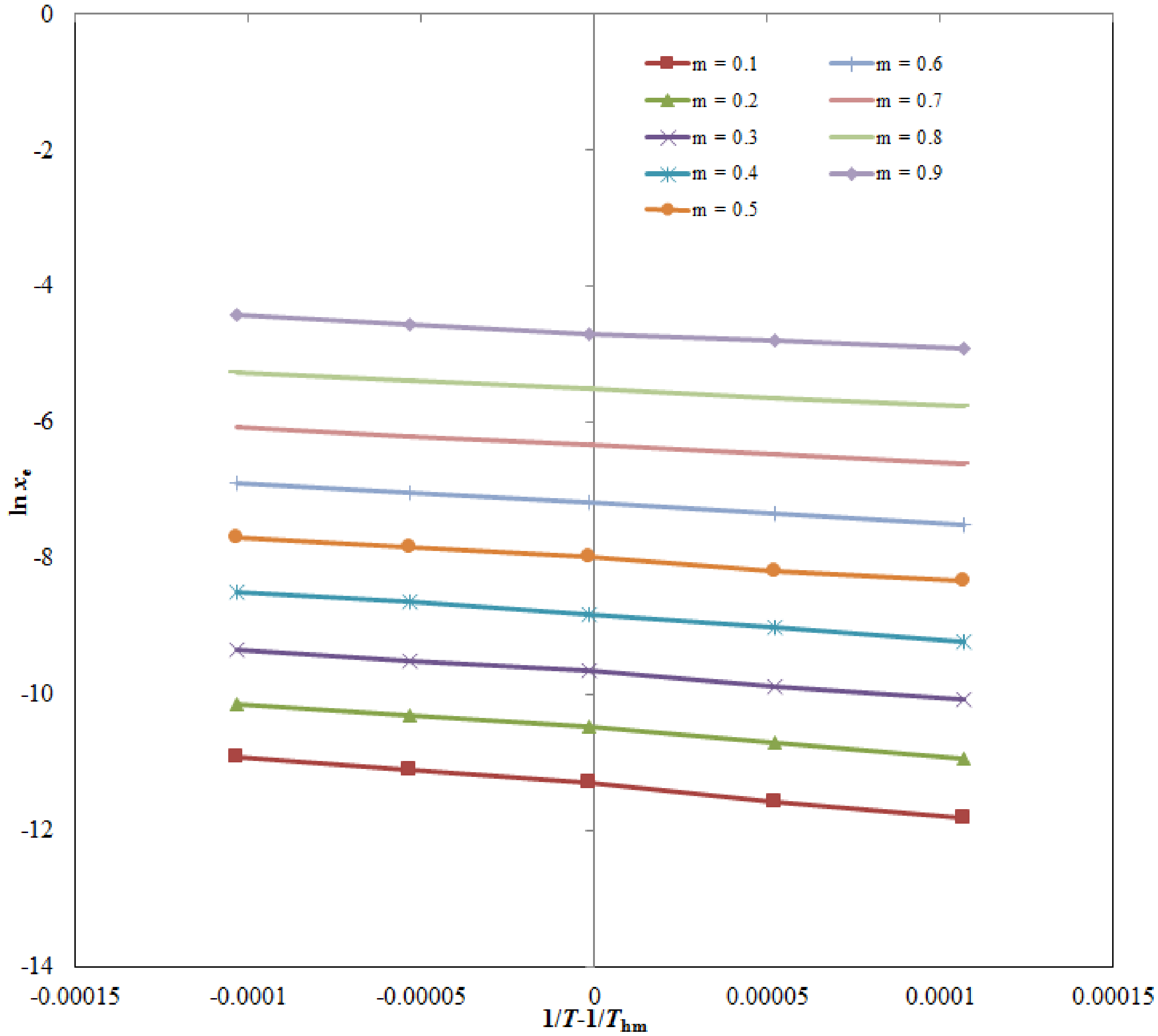

Figure S4. van't Hoff plots for TRV plotted between ln x_e and 1/T-1/T_{hm} for TRV in various PG + water mixtures.

Table S1. Hansen solubility parameters ($\delta_{mix}/MPa^{1/2}$) for various PG + water mixtures free of TRV at 298.2 K.

m	$\delta_{mix}/MPa^{1/2}$
0.1	45.94
0.2	44.08
0.3	42.22
0.4	40.36
0.5	38.50
0.6	36.64
0.7	34.78
0.8	32.92
0.9	31.06

Table S2. Details information about materials used.

Materials	Molecular formula	Molar mass (g mol^{-1})	CAS Registry no.	Purification method	Mass fraction purity	Analysis method	Source
TRV	$C_{14}H_{12}O_3$	228.24	501-36-0	None	0.993	HPLC	Sigma Aldrich
Methanol	CH_3OH	32.04	67-56-1	None	0.994	GC	Alfa Aesar
Ethanol	C_2H_5OH	46.07	64-17-5	None	0.992	GC	Alfa Aesar
n-Propanol	C_3H_8O	60.10	71-23-8	None	0.993	GC	Alfa Aesar
n-Butanol	$C_4H_{10}O$	74.12	71-36-3	None	0.992	GC	Alfa Aesar
PG	$C_3H_8O_2$	76.09	57-55-6	None	0.994	GC	E-Merck
Water	H_2O	18.07	7732-18-5	None	-	-	Milli-Q

Both the method of analysis and purity of materials were provided by the supplier of each material.
